# Potent hERG channel inhibition by sarizotan, an investigative treatment for Rett Syndrome

**DOI:** 10.1016/j.yjmcc.2019.07.012

**Published:** 2019-10

**Authors:** Hongwei Cheng, Chunyun Du, Yihong Zhang, Andrew F. James, Christopher E. Dempsey, Ana P. Abdala, Jules C. Hancox

**Affiliations:** a School of Physiology, Pharmacology and Neuroscience, Biomedical Sciences Building, University Walk, Bristol BS8 1TD, United Kingdom; b School of Biochemistry, Biomedical Sciences Building, University Walk, Bristol BS8 1TD, United Kingdom

**Keywords:** hERG, KCNH2, Long QT syndrome, Rett Syndrome, Sarizotan

## Abstract

Rett Syndrome (RTT) is an X-linked neurodevelopmental disorder associated with respiratory abnormalities and, in up to ~40% of patients, with prolongation of the cardiac QT_c_ interval. QT_c_ prolongation calls for cautious use of drugs with a propensity to inhibit hERG channels. The STARS trial has been undertaken to investigate the efficacy of sarizotan, a 5-HT_1A_ receptor agonist, at correcting RTT respiratory abnormalities. The present study investigated whether sarizotan inhibits hERG potassium channels and prolongs ventricular repolarization. Whole-cell patch-clamp measurements were made at 37 °C from hERG-expressing HEK293 cells. Docking analysis was conducted using a recent cryo-EM structure of hERG. Sarizotan was a potent inhibitor of hERG current (I_hERG_; IC_50_ of 183 nM) and of native ventricular I_Kr_ from guinea-pig ventricular myocytes. 100 nM and 1 μM sarizotan prolonged ventricular action potential (AP) duration (APD_90_) by 14.1 ± 3.3% (*n* = 6) and 29.8 ± 3.1% (*n* = 5) respectively and promoted AP triangulation. High affinity I_hERG_ inhibition by sarizotan was contingent upon channel gating and intact inactivation. Mutagenesis experiments and docking analysis implicated F557, S624 and Y652 residues in sarizotan binding, with weaker contribution from F656. In conclusion, sarizotan inhibits I_Kr_/I_hERG_, accessing key binding residues on channel gating. This action and consequent ventricular AP prolongation occur at concentrations relevant to those proposed to treat breathing dysrhythmia in RTT. Sarizotan should only be used in RTT patients with careful evaluation of risk factors for QT_c_ prolongation.

## 1 Introduction

Rett Syndrome (RTT) is a severe X-chromosome-linked developmental disorder characterized by cognitive and motor skill deficits, together with autistic spectrum features, seizures and microencephaly [[1], [2], [3]]. The condition is strongly associated with autonomic dysfunction, manifested in respiratory difficulties (including hyperventilation, breath-holding and air swallowing) and abnormal heart rate control [1,3]. Patients with RTT are almost exclusively female; males exhibit more severe respiratory and heart rate abnormalities and most die within a year of birth [2]. RTT is usually caused by mutations in the X-linked transcriptional regulator gene that encodes methyl-CpG-binding protein 2 (MECP2) [2,[4], [5], [6], [7]]. *MECP2* gene mutations were identified in >95% of individuals with RTT [8]. Approximately 26% of deaths in RTT are sudden and unexpected [9]. A proportion of RTT patients exhibit prolongation of the rate-corrected QT (QT_c_) interval and T wave abnormalities [[10], [11], [12], [13], [14]]^.^, which appear to be independent of electrolyte abnormalities [11]. In 1994 Sekul and colleagues reported lengthened QT_c_ intervals and T wave abnormalities in 41% of girls with RTT compared to age-matched healthy controls [10]. The proportion of QT interval/T wave abnormalities increased with increasing severity of disease [10]. A subsequent study of a cohort of 34 RTT patients showed a prolonged QT_c_ interval in 9 patients and an upper borderline value in 10 patients; QT_c_ prolongation was present in the absence of electrolyte abnormalities [11]. Examination of 74 females with RTT syndrome and 10 with an atypical RTT variant with preserved speech found QT_c_ prolongation in 55% of girls with classic RTT compared to 20% with the atypical variant [12].

Similar to humans, both mouse and primate models of *MECP2* linked RTT manifest QT_c_ interval prolongation [[13], [14], [15], [16]]. Experiments on 2–3 month old *Mecp2*^null/Y^ mice have revealed these to exhibit longer QT_c_ intervals than those from wild-type (WT) mice, in the absence of any obvious structural or contractile abnormality [13]. Whole-cell patch-clamp recordings from isolated ventricular myocytes from *Mecp2*^null/Y^ mice showed no change in peak sodium current (I_Na_) elicited on step depolarization to −20 mV, but showed an increased persistent, late sodium current component, I_Na,Late_ [13]. Thus, prolonged QT_c_ intervals in RTT may result from enhanced ventricular I_Na,Late_ [13].

There are currently no specific treatments for breathing abnormalities in RTT. However, in mouse models of RTT, the investigational drug sarizotan (5-HT_1A_ agonist, D_2_ partial agonist) has been found to correct irregular breathing patterns and reduce the incidence of apnoeas [17]. The STARS (**S**arizotan **T**reatment of **A**pneas in **R**ett **S**yndrome) double-blind, placebo-controlled clinical study [18] is investigating tolerability of sarizotan for apnoea treatment in RTT patients. As RTT is associated with QT_c_ interval prolongation, it is important that drug treatments for respiratory, cognitive or motor deficits in the syndrome do not exacerbate this phenotype, as QT_c_ prolongation carries a risk of *Torsades de Pointes* (TdP) arrhythmia and sudden death [19,20]. Virtually all drugs associated with QT_c_ prolongation inhibit potassium channels encoded by *hERG* (*human Ether-à-go-go Related Gene;* alternative nomenclature *KCNH2*), which mediate the cardiac rapid delayed rectifier K^+^ current, I_Kr_ [20,21]. There are currently no published studies addressing the issue as to whether or not sarizotan inhibits the hERG channel. The present study was undertaken to address this. It demonstrates that sarizotan produces potent inhibition of hERG K^+^ channel current (I_hERG_) and prolongs ventricular repolarization, findings of critical importance for the deployment of the drug in the RTT patient population.

## 2 Methods

### 2.1 Wild-type and mutant hERG channels

Human Embryonic Kidney (HEK293) cells stably expressing wild-type (WT) hERG were generously donated by Dr. Craig January, University of Wisconsin [22]. A cell line stably expressing Y652A hERG was created in the laboratory by Milnes et al. [23]. hERG mutations were constructed using QuikChange site-directed mutagenesis (Stratagene, La Jolla, CA) as previously reported [24,25]. The complementary oligonucleotide primers used for F656 V mutation construction were: Forward 5′- GTATGCTAGCATCGTCGGCAACGTGTCG −3′ and Reverse 5’-CGACACGTTGCCGACGATGCTAGCATAC-3′. All the mutations were confirmed by entire open reading frame sequencing (Eurofins MWG Operon, Ebersberg, Germany).

### 2.2 Cell maintenance and transfection

Cells were maintained and passaged as described previously [24,26,27]. Transient transfections were conducted when the cells reached >80% confluence, using Lipofectamine 2000 (Life Technologies, Carlsbad, CA) according to the manufacturer's instructions. Expression plasmid encoding CD8 (in pIRES, donated by Dr. I Baró, University of Nantes, France) was also transfected. Successfully transfected cells were detected using Dynabeads® (Invitrogen, Paisley, UK). Cells were incubated at 37 °C for a minimum of 24 h prior to any electrophysiological recording.

### 2.3 Guinea-pig ventricular myocyte isolation

Guinea-pig ventricular myocytes have utility in evaluating the consequences of hERG channel blocking drugs [26,28]. Left ventricular myocytes from guinea-pig heart were isolated by enzymatic and mechanical dispersion as described previously [26,29,30]. Male Dunkin Hartley Guinea-pigs (Marshall BioResources) were killed in accordance with UK Home Office legislation. Briefly, guinea-pigs (300-600 g) were terminally anesthetized with pentobarbital sodium (140 mg/kg, I.P.) together with heparin (4000 U/kg). The heart was removed quickly and was then cannulated and perfused via a Langendorff perfusion system at 37 °C. The basic perfusion solution contained (mM): 130 NaCl, 5.4 KCl, 5 HEPES, 10 Glucose, 0.4 NaH_2_PO_4_, 3 MgCl_2_, 20 taurine and 10 creatine (pH 7.61 with NaOH). First, this basic solution with added CaCl_2_ (750 μM) was perfused for 2 min, followed by the basic solution for 5 min [29]. This was followed by low Ca^2+^ (150 μM) solution containing collagenase (Type I, Worthington 0.3 mg/ml per 100g body weight) and protease (Type XIV, Merck 0.01 mg/ml per 100g body weight) for 4–7 min. Cells were then released from left ventricle by mechanical dispersion. Isolated myocytes were stored in low calcium solution (150 μM) at room temperature. For recording, an aliquot of the cell suspension was transferred into a chamber mounted on the microscope and left to settle for several minutes, before being exposed to normal Tyrode solution. Only cells with clear rod-shape and striated appearance were chosen for recording.

### 2.4 Electrophysiological recordings

Whole-cell patch-clamp experiments were performed using an Axopatch-1D amplifier and Digidata 1322A or 1440A (Molecular Devices, USA). Protocols were generated, and data recorded online with pClamp 8.0 or 10.0 (Molecular Devices, USA). The digitization rate was 10–20 kHz. Patch-pipettes (A-M Systems, USA) were pulled using a vertical electrode puller (Narishige PP-83, Japan), and heat-polished to a final resistance of 2–3 MΩ (Narishige MF-83, Japan). Capacitance and series resistance were routinely compensated, with series resistance compensation of ~70%. Conventional whole-cell patch-clamp recordings were made to measure hERG current (I_hERG_) from HEK 293 cells expressing WT or mutant hERG channels, and native delayed rectifier potassium current (I_Kr_) from guinea-pig ventricular myocytes. Guinea-pig action potentials (APs) were recorded using perforated-patch with 400 μg/ml amphotericin B in pipette solution. All measurements were performed at 37 °C.

**Fig. 1:**
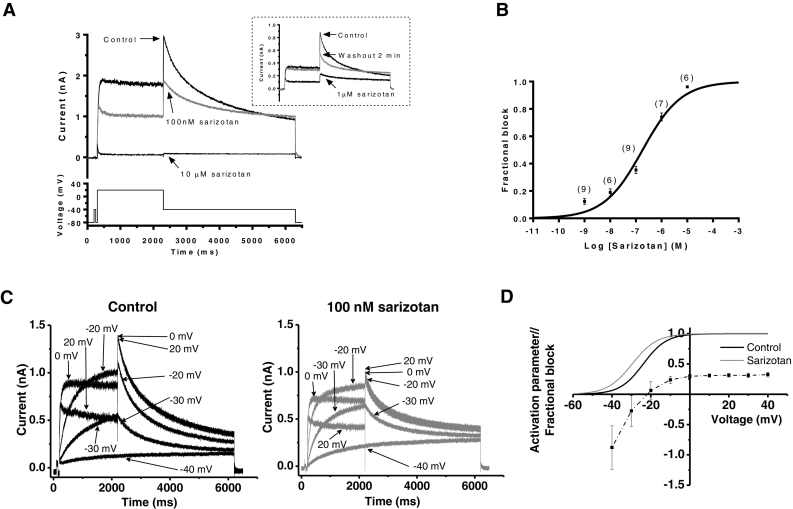
(A) Upper traces show I_hERG_ in control solution and in the presence of 100 nM and 10 μM sarizotan; voltage protocol shown as lower trace. Inset shows recordings from a separate experiment, showing partial reversibility of 1 μM sarizotan. Cross-over of washout and control I_hERG_ tails is consistent with ‘foot-in-the door’ inhibition. (B) Concentration-response relation for I_hERG_ inhibition. Mean fractional block of I_hERG_ is shown plotted against corresponding drug concentration. Replicate numbers at each concentration shown in brackets. IC_50_ and n_H_ values given in “Results” text. (C) Representative traces of I_hERG_ in control (black) and 100 nM sarizotan (grey) at selected potentials during protocol like that in A, but with test potentials between −40 and +40 mV. (D) Mean fractional block of I_hERG_ tails by 100 nM sarizotan plotted against test voltage (*n* = 7). Activation relations for control and sarizotan are shown superimposed (V_0.5_ and *k* values in the main text).

**Fig. 2:**
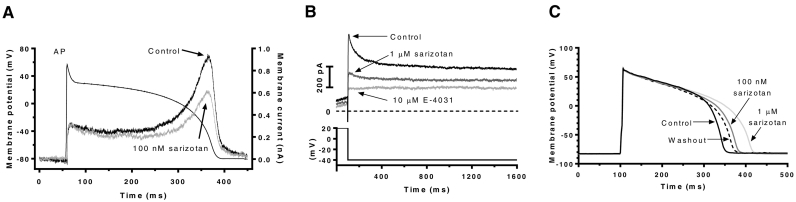
(A) Representative traces of I_hERG_ in control and 100 nM elicited by ventricular AP clamp (AP shown superimposed). (B) Representative traces of guinea-pig ventricular deactivating I_Kr_ tails (elicited on repolarization to −40 mV following 500 ms command to +20 mV) in control, 1 μM sarizotan and 10 μM E-4031. Sarizotan was applied in these experiments for 3–5 min and 1 μM E-4031 for 1 min. (C) Ventricular APs (elicited at 0.1 Hz) in control solution, after 100 nM and 1 μM sarizotan and following washout. Mean AP duration at 25% and 90% repolarization (APD_25_ and APD_90_) values are given in the main text.

**Fig. 3:**
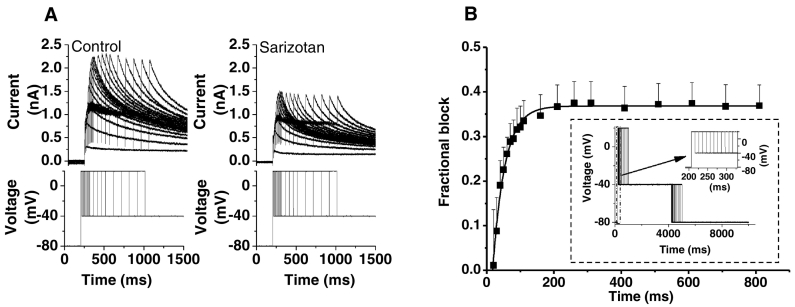
(A) Upper traces show representative current traces elicited by the envelope of tails protocol shown in as lower traces. Left hand panel shows data in Control, right hand panel shows data in 100 nM sarizotan. (B) Mean (*n* = 5) I_hERG_ blocking time-course at +20 mV derived from application of the envelope of tails protocol. Protocol was applied in control, cells then rested in sarizotan for 3–5 min, and protocol was reapplied to ascertain fractional block values. The inset shows the protocol with a high gain insert to show more clearly the steps of short duration early during the protocol. Fractional block data at different time-points were fitted with an exponential to ascertain the time constant value given in the Results text.

**Fig. 4:**
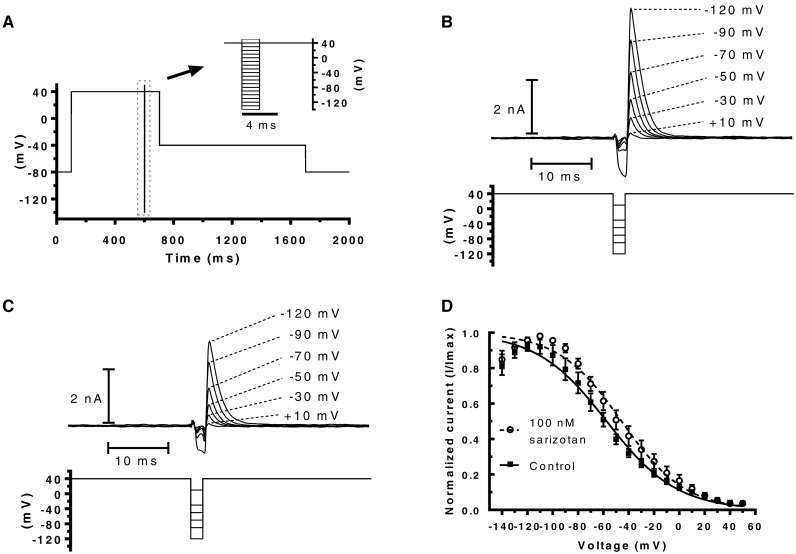
(A) Protocol used to investigate I_hERG_ ‘availability’. From a − 80 mV, the membrane potential was stepped to +40 mV for 500 ms; this was then followed by 2 ms repolarization steps to potentials ranging from −140 mV to +50 mV, membrane potential was then stepped back to +40 mV for 100 ms (inset shows portion of protocol encompassing the transition to and from repolarizing steps). The amplitude of current transients elicited by the second step to +40 mV was used to assess I_hERG_ availability. (B) Representative traces in control. Selected traces are shown for clarity. Numbers indicate corresponding voltage of preceding 2 ms repolarization step. (C) Representative traces at 100 nM sarizotan. (D) Plots against voltage of normalized current transient amplitude in control and in 100 nM sarizotan (n = 7 cells); the inactivation V_0.5_ and *k* values were derived from fits with standard Boltzmann function and are given in the Results text.

A standard voltage-protocol (lower trace of Fig. 1A) was applied from a holding potential of −80 mV to measure I_hERG._ The protocol incorporated a brief (50 ms) pre-pulse from −80 to −40 mV prior to the +20 mV test command, in order to quantify the instantaneous current at −40 mV. Comparison between this instantaneous current and the peak outward I_hERG_ tail amplitude on repolarization to −40 mV enabled the accurate measurement of I_hERG_ tail amplitude [31,32]. The sweep start-to-start interval was 12 s. The protocol used to investigate voltage-dependence of inhibition of I_hERG_ by sarizotan was like that shown in Fig. 1A, but with test voltages between −40 and +40 mV. Action potential voltage clamp (AP clamp) experiments employed a human epicardial ventricular AP waveform (Fig. 2A) from the ten Tusscher *et al* ventricle model [33] identical to that used in prior experiments from our laboratory (e.g. [31]). AP commands were applied every 3 s and online leak subtraction was performed using a P/4 protocol [34]. An ‘envelope of tails’ protocol (lower traces in Fig. 3A) was used to investigate the development of inhibition of I_hERG_ by sarizotan with time following membrane depolarization. From holding of −80 mV, cell membrane potential was depolarized to +20 mV in steps of incrementing duration from 10 ms to 810 ms; the amplitude of I_hERG_ tails on repolarization to −40 mV reflected the extent of I_hERG_ activation produced during the pulses to +20 mV. The protocol used to investigate I_hERG_ ‘availability’ is shown in Fig. 4A. From −80 mV, the membrane potential was stepped to +40 mV for 500 ms; this was then followed by 2 ms repolarization steps to potentials ranging from −140 to +50 mV; membrane potential was then stepped back to +40 mV for 100 ms. The amplitude of current transients elicited by the second step to +40 mV was used to assess I_hERG_ availability. The protocol to measure I_Kr_ was comprised of a brief step from −80 to −40 mV (to inactivate fast Na channels), followed by a 500 ms step to +20 mV and repolarization to −40 mV to observe I_Kr_ tails (lower trace of Fig. 2B) [35]. The sweep start-to-start interval was 10 s. Action potentials from guinea-pig myocytes were elicited in current-clamp mode, by brief (5 ms) duration suprathreshold depolarizing current pulses at a stimulation frequency of 0.1 Hz.

### 2.5 Computational docking

Computational docking was conducted using the open pore cryo-EM structure of hERG [PDB: 5VA1] [36], as recently described [27] using GOLD following the methods described in [37]. Free side chain flexibility was allowed during docking for selected residues within the potential drug binding site within the hERG pore to accommodate and optimize drug interactions. The potential binding site was centred above the β−carbon of the chain-A Tyr-652 side chain and a radius of 10 or 12 Å was selected to allow the sarizotan molecule to sample configurational space within the chain A hydrophobic pocket and also the surrounding parts of the hERG pore. Due to the large number of rotamers sampled during docking 300,000 generations of the genetic algorithm was used; the ChemPLP scoring function was used to select low energy score docking poses from a set of 100 docking runs for each of the sarizotan enantiomers.

### 2.6 Data analysis

Data are presented as mean ± SEM. Statistical analysis and fits to data sets were performed using Microsoft Excel (Microsoft Corporation, USA), Origin 7.0 (OriginLab, USA), Prism 7.04 (Graphpad Software Inc., USA) and Clampfit of pClamp 10.7 (Molecular Devices, USA). Data distribution was tested using the Kolmogorov–Smirnov normality test. Comparisons were made using paired *t*-test, unpaired *t*-test; Wilcoxon signed rank test, or one-way ANOVA followed by a Tukey post-test as appropriate; *P* < .05 was taken as significant. The following standard eqs. [24,26] were used to fit particular data-sets:

Fractional block of I_hERG_ was determined using an equation of the form:

(1) $$\mathrm{Fraction}\;\mathrm{block}=1–\left(I_{\mathrm{hERG}}\;\mathrm{residual}/I_{\mathrm{hERG}}\;\mathrm{control}\right)$$

where I_hERG_ control represents current amplitude prior to drug application and I_hERG_ residual represents the unblocked current remaining following drug exposure.

Concentration-response data were fitted with a Hill equation of the form:

(2) Fractionalblock=1/1+10^LogIC50−X∗h

where Fractional block refers to the degree of inhibition of I_hERG_ by a given concentration of sarizotan (X, the logarithm of concentration); IC_50_ is [sarizotan] producing half-maximal inhibition of I_hERG_, and *h* is the Hill coefficient for the fit.

Voltage-dependent activation parameters were derived from current-voltage (I-V) relations for I_hERG_ tail currents, fitted with a Boltzmann equation of the form:

(3) $$I=I_{\max}/\left(1+\exp\left(\left(V_{0.5}-V_{m}\right)/k\right)\right)$$

where I is tail current magnitude at −40 mV following a voltage test potential (V_m_); I_max_ is the maximal tail current during the voltage protocol; V_0.5_ is the voltage at which I_hERG_ is half maximally activated, and *k* is the slope factor for the relation.

Continuous plots of I_hERG_ activation were produced by inserting the values for V_0.5_ and *k* obtained from Eq. (3) into the equation:

(4) $$\mathrm{Activation}\;\mathrm{variable}=1/\left(1+\exp\left(\left(V_{0.5}-V_{m}\right)/k\right)\right)$$

where the terms have similar meanings to those described above for Eq. (3).

I_hERG_ blocking time-course from application of the envelope of tails protocol was fitted with a single-exponential equation:

(5) $$\mathrm{Fractional}\;\mathrm{block}=Y_{0}+\left(\mathrm{Plateau}-Y_{0}\right)*\left(1-\exp\left(-x/\tau \right)\right)$$

where Fractional block refers to the degree of inhibition of I_hERG_ at a pulse duration ‘x’; the fractional block starts at Y_0_, then goes to Plateau; and τ is the time constant of development of I_hERG_ inhibition.

Voltage dependence of I_hERG_ availability (inactivation) was determined by fitting normalized current transients with the equation:

(6) $$\left(I/I_{\max}\right)=1–1/\left(1+\exp\left(\left(V_{0.5}-V_{m}\right)/k\right)\right)$$

where the normalized current (I/I_max_) is a ratio of a current (I) at a membrane potential/voltage (V_m_) and the maximal current (I_max_) during the voltage protocol (−140 to +50 mV); V_0.5_ is the potential at which I_hERG_ was half maximally inactivated, and *k* is the slope factor for the relation.

## 3 Results

### 3.1 Potency and voltage dependence of sarizotan inhibition of I_hERG_

Sensitivity of I_hERG_ to sarizotan was evaluated using a previously described voltage protocol (lower trace in Fig. 1A) [23,24]. In the example shown the I_hERG_ tail was inhibited by 37% at 100 nM and by 96% at 10 μM sarizotan. The inset shows partial reversibility of the response to sarizotan (1 μM). A total of 5 concentrations were tested and a mean concentration-response plot was constructed (Fig. 1B), yielding an IC_50_ of 183 ± 51 nM, with a Hill co-efficient (n_H_) of 0.58 ± 0.10. Sarizotan action on I_hERG_ tails following different test potentials was assessed using a similar protocol, but with test voltages between −40 and + 40 mV. Representative current traces are shown in Fig. 1C. I_hERG_ tail block by 100 nM sarizotan was evaluated following the different command voltages and plotted as shown in Fig. 1D. Tail currents in control and drug following the different test potentials were fitted with a Boltzmann function and mean derived V_0.5_ and *k* values then used to construct activation plots, superimposed in Fig. 1D. Voltage dependent activation was leftward shifted in sarizotan compared to control (V_0.5_ in sarizotan of −29.9 ± 2.3 mV and in control of −24.2 ± 1.9 mV, *P* < .05; *k* in sarizotan of 6.1 ± 0.8 and in control 5.7 ± 0.3, *P* > .84, *n* = 7; Wilcoxon matched-pairs signed rank test for both). The progressive changes in extent of I_hERG_ block by sarizotan coincided with the steep phase of I_hERG_ activation, consistent with activation-dependent inhibition [26]. Over potentials (between +10 and + 40 mV) at the top of the voltage-dependent activation relation there was no significant change in the extent of fractional inhibition, suggesting that voltage-dependence of inhibition independent of channel gating did not occur.

### 3.2 Effects of sarizotan on ventricular action potentials and I_Kr_

Fig. 2A shows effects of 100 nM of sarizotan on I_hERG_ elicited by a ventricular AP waveform. Peak repolarizing current occurred at −38.6 ± 1.6 mV in control and − 35.2 ± 2.7 mV in sarizotan (*P* > .05, paired *t*-test; *n* = 7). It was reduced by 33.0 ± 3.2% in sarizotan (*P* > .05, cf standard protocol (35.4 ± 2.6%; *n* = 9) unpaired t-test). We evaluated block of native ventricular I_Kr_ using a protocol comprised of a brief step from −80 to −40 mV (to inactivate fast Na channels), followed by a 500 ms step to +20 mV and repolarization to −40 mV to observe I_Kr_ tails. 1 μM sarizotan suppressed deactivating tail current amplitude by 66.2 ± 1.9% (n = 7). As shown in Fig. 2B, with subsequent application of a high concentration of E-4031 after sarizotan there was no residual tail current, confirming that under these conditions deactivating outward current tails were carried by I_Kr_. We also monitored the peak inward L-type Ca^2+^ current (I_Ca,L_) at the start of the +20 mV step of this protocol and observed only a small (18.4 ± 3%; n = 7) reduction in that current with 1 μM sarizotan (not shown). Fig. 2C shows the effect of 100 nM and 1 μM sarizotan on ventricular AP duration (APD). 100 nM sarizotan prolonged action potential duration at 25% repolarization (APD_25_) by 3.1 ± 1.8% and action potential duration at 90% repolarization (APD_90_) by 14.1 ± 3.3% (*n* = 6), whilst 1 μM sarizotan prolonged APD_25_ by 3.8 ± 1.2% and APD_90_ by 29.8 ± 3.1% (*n* = 5). AP triangulation [38] was measured as the APD_25_ to APD_90_ difference. This was 100.7 ± 7.6 ms in control, 126.4 ± 10.7 ms in 100 nM sarizotan and 173.9 ± 9.9 ms in 1 μM sarizotan (*P* < .01 vs control for both; one-way ANOVA with Tukey post-test). Significant triangulation was also observed when measured as APD_30_-APD_90_ difference (see Table 1). Thus, sarizotan both prolonged ventricular APD and increased AP triangulation.

**Table 1:** Effects of sarizotan exposure on ventricular AP repolarization parameters.

	Control (*n* = 6)	Sarizotan 100 nM (n = 6)
APD_25_ (ms)	118.4 ± 17.7	121.4 ± 17.8
APD_30_ (ms)	137.9 ± 20.1	142.9 ± 19.4
APD_50_ (ms)	189.1 ± 21.9	204.7 ± 23.9 ⁎⁎
APD_60_ (ms)	200.4 ± 21.6	224.3 ± 21.3 ⁎⁎
APD_90_ (ms)	219.0 ± 21.7	247.8 ± 21.3 ⁎⁎
APD_90_-APD_25_ (ms)	100.6 ± 9.0	126.4 ± 10.7 ⁎⁎
APD_90_-APD_30_ (ms)	81.1 ± 7.4	105.0 ± 9.5 ⁎⁎

APD refers to action potential duration and subscripted numbers refer to point after start of repolarization at which duration was measured (values are given for 25%, 30%, 50%, 60%, 90% of complete repolarization).

⁎⁎ denotes statistical significance of *P* < .01 versus control; paired *t*-test.

### 3.3 Time dependence and effect of sarizotan on I_hERG_ inactivation

Time-dependence of I_hERG_ block was explored using two complementary approaches. The first of these utilized a protocol comprised of a long (10 s) duration depolarization from −80 to 0 mV [39], which was applied first in control solution and then in 100 nM sarizotan, after resting the cell at −80 mV in sarizotan for ~3 min (representative traces are shown in supplemental Fig. S1A). As shown in Fig. S1B, a substantial component of the block observed at 10 s at 0 mV occurred by 100–200 ms into the depolarizing command, indicating that block was evident quickly on membrane depolarization. However, as highlighted previously in this journal, this protocol is less suited to examination of block over comparatively shorter than longer periods following membrane depolarization [40]. Consequently, we also employed an envelope of tails protocol (with depolarizing commands to +20 mV of differing duration, shown as lower traces in Fig. 3A, with representative currents shown as the upper traces) [35,40,41]. This was applied in control, the cell then rested for ~3 min during exposure to 100 nM sarizotan, and then reapplied. Fractional inhibition of the tail currents following the different duration voltage commands was then determined and plotted against test pulse duration as shown in Fig. 3B (*n* = 5). There was little or no block with very short voltage commands, and block increased progressively over ~200 ms. A single exponential fit to these data gave a blocking τ of 34.4 ms. These results demonstrate that sarizotan's action was contingent upon channel gating with little or no block of resting channels.

Fig. 4A shows the protocol used to evaluate voltage dependent availability of I_hERG_, from which inactivation V_0.5_ and *k* values were obtained [42,43]. Fig. 4B and C respectively show representative traces, focusing on the portion of the protocol including brief repolarizing steps (to produce differing extents of I_hERG_ availability) and the subsequent outward I_hERG_ transients during the final step of the protocol. Plots of I_hERG_ availability (Fig. 4D) were constructed as described previously [42] and Boltzmann fits used to obtain inactivation V_0.5_ and *k* values (V_0.5_ in sarizotan of −45.3 ± 5.0 mV and in control of −57.9 ± 4.5 mV; *P* > .05, *n* = 7 paired *t*-test; *k* in sarizotan of 23.1 ± 1.1 and in control of 26.3 ± 1.9, *P* > .05, n = 7; paired *t*-test). Time-course of inactivation was evaluated for current following a brief conditioning pulse to −120 mV and was not significantly altered by sarizotan (τ values of 1.74 ± 0.43 and 1.93 ± 0.22 ms in control and sarizotan respectively; *P* > .05; paired *t*-test; data not shown).

### 3.4 Molecular basis of sarizotan action

**Fig. 5:**
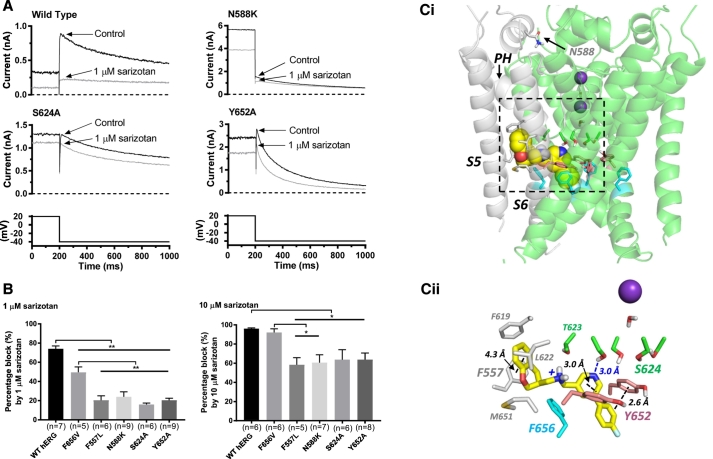
(A) Representative traces showing effects of sarizotan on I_hERG_ tail current for WT, N588K, S624A and Y652A hERG channels (shown as upper sets of traces) with expanded portion of voltage protocol shown as lower traces. (B) Bar charts showing mean fractional block of I_hERG_ tails by 1 μM and 10 μM sarizotan for WT, N588K, S624A, F557 L, Y652A and F656A hERG channels. Replicate numbers shown in brackets. * and ** are significance at *P* < .05 and *P* < .01 respectively (one-way ANOVA with Tukey post-test). (Ci) Low energy score configuration for R sarizotan (yellow space filling representation) docked into the cryo-EM structure of hERG [36]. Subunit A of the hERG pore is colored grey and the side chains shown are identified in Cii. The location of N588 in chain A is also shown. The selectivity filter is occupied by K^+^ ions in the 1 and 3 positions (purple spheres) and waters in the 2 and 4 positions. The S enantiomer of sarizotan (not shown) was similarly able to bind with a hydrophobic aromatic part of the drug projecting into a hydrophobic pocket. PH: pore helix. (Cii) Close up of the bound configuration for R sarizotan as in panel Ci showing side chains in the binding “pocket” [36] of chain A (grey sticks) and other side chains important for binding to hERG blockers and mentioned in the text. (For interpretation of the references to colour in this figure legend, the reader is referred to the online version of this article.)

The effects of targeted hERG mutations on inhibition of I_hERG_ by 1 μM and 10 μM sarizotan action were established. The N588K mutation to the S5-Pore linker region markedly reduces I_hERG_ inactivation and attenuates blockade of a range of agents that rely on intact inactivation to bind to the channel [32,44]. I_hERG_ inhibition by both 1 μM and 10 μM sarizotan was significantly attenuated by this mutation, indicating that an intact inactivation process is needed for high affinity sarizotan binding. Representative traces for the S624A mutation (located in the pore helix/base of selectivity filter) and Y652A mutation (located in the S6 helix) are also shown in Fig. 5A, with attenuation of block by 1 μM sarizotan. Fig. 5B summarises effects of these mutations plus S6 F656 V and S5 F557 L mutations, both of which also attenuated drug action. Supplemental Table S1 provides estimates of the changes in binding energy for sarizotan introduced by the mutations studied.

Fig. 5 Ci and 5Cii show results of *in silico* docking of sarizotan within the pore of the recently determined cryo-EM hERG channel structure [36]. Sarizotan could bind within the pore below the selectivity filter, with a hydrophobic aromatic part of the drug projecting into one of the hydrophobic pockets identified in the EM structure that are found at the interface between the pore helix (PH) and S5 and S6 helices (Fig. 5 Ci). In this configuration sarizotan made π-π stacking interactions with F557 and Y652 aromatic side chains, and the polar amino and pyridine nitrogens lay below the C-terminal end of the pore helix with possible direct or water-mediated interactions with the T623 and S624 hydroxyl side chains (Fig. 5Cii); interactions with F656 in this configuration were minimal. This docking pose is consistent with the mutagenesis data summarized in Fig. 5B. The specific binding interactions between sarizotan and S624, Y652 and F557 are further highlighted in the stereo view in supplemental Fig. S2.

## 4 Discussion

### 4.1 Sarizotan interaction with the hERG channel

This study demonstrates that sarizotan inhibits both I_hERG_ and native I_Kr_ at sub-micromolar concentrations (IC_50_ for I_hERG_ block of 183 nM), prolongs ventricular APD_90_ and increases ventricular AP triangulation, which is a marker of proarrhythmic risk [38]. The observed potency of sarizotan inhibition of I_hERG_ can usefully be considered in the context of IC_50_ values obtained in our laboratory under similar experimental conditions for established I_hERG_ inhibitors. Thus, using the standard protocol shown in Fig. 1A, we have previously obtained I_hERG_ IC_50_ values for the canonical hERG/I_Kr_ blockers E-4031 and dofetilide of 16 nM and 9.8 nM respectively and for the Class Ia antiarrhythmic quinidine of 620 nM [32,45]. Both dofetilide and quinidine are well established to be linked to QT_c_ interval prolongation [19] and it is notable that sarizotan is a more potent inhibitor of I_hERG_ than is quinidine under similar experimental conditions.

The WT I_hERG_ data in this study show that sarizotan relies on hERG channel gating in order to access its binding site, whilst the N588K mutant data demonstrate that an intact inactivation process is necessary for high affinity binding [32;^44^]. High affinity hERG channel inhibition most commonly involves drug interaction with S6 aromatic residues, with further supportive interactions with other residues on the S6 helices and located close to the selectivity filter/base of the pore helix [21;^46^]. Our mutagenesis and docking results indicate that sarizotan interacts with residues of the canonical drug binding site, though it is notable that mutation at Y652 led to a greater attenuation of inhibition than did mutation of F656. Recent studies have shown a role for the F557 S5 aromatic residue in I_hERG_ block [27;^47^] and, here, mutation of F557 markedly reduced I_hERG_ block by sarizotan. F557 lies partly within hydrophobic pockets recently identified in the cryo-EM structure of hERG [36]. Docking to this structure shows low-energy binding could occur in a configuration that places sarizotan near F557, S624 at the base of the selectivity filter and Y652. The original study that identified F557 as a potential binding determinant for hERG inhibitors did not directly compare effects of mutations at F557 and F656 experimentally [47]. In our prior study of a minimally structured high affinity hERG inhibitor, Cavalli-2, mutation at F656 had greater effects on blocking potency than mutation at either F557 or Y652 [27]. An independent study of a novel EGFR inhibitor FHND004 also found mutation at F656 to attenuate inhibition more than at F557 [48]. Sarizotan is notable in this regard as it is an I_hERG_ inhibitor for which mutation of F557 produces a greater attenuation of inhibition than mutation at the canonical F656 binding residue. Interestingly, a very recent independent study of the HCN channel inhibitor ivabradine has reported I_hERG_ inhibition by that drug to be attenuated more by the F557L than F656C mutation [49].

### 4.2 Implications of the study

The STARS trial involves administration of 2 or 10 mg sarizotan bidaily (over 24 weeks) to RTT patients aged ≥4 years, with a body weight of ≥10 kg, with a primary outcome measure of reduction of respiratory abnormalities [18]. Redfern *et al* proposed a safety margin of 30 (hERG IC_50_/ plasma C_max_) for drugs undergoing clinical evaluation [50], which would correspond to a sarizotan plasma level of 6–7 nM (for the I_hERG_ IC_50_ in this study). Oral administration of 2 and 10 mg of sarizotan to adult men produced peak total plasma concentrations of ~170 and 488 ng/ml (~ 442 nM and 1.27 μM, respectively [51]). Therefore, it is expected that peak total plasma levels of sarizotan in young RTT patients given 2 or 10 mg doses may fall within a comparable range. Although unbound levels may be substantially lower than total plasma drug levels, it is notable that sarizotan is lipophilic (logP value of 4.1; PubChem CID 6918388) and so could concentrate in lipid membranes. Feasibly, therefore, sarizotan administration could result in a safety margin of <30. Thus, it is reasonable to conclude that hERG/I_Kr_ block is a potential risk of sarizotan administration to RTT patients. This would pose a particular safety concern in the subset of RTT patients with overt QT_c_ prolongation [[10], [11], [12], [13], [14]]. Administration of sarizotan to primates is associated with production of metabolites (EMD 148107, EMD 329989, EMD 50929) at concentrations 5–10 fold lower than that of the parent compound [52]. The propensity of these metabolites to inhibit hERG/I_Kr_ and delay repolarization remains to be established. Exclusion criteria for the STARS trial include a Fridericia corrected QT_c_ interval of >450 ms. However, in principle, individuals with a borderline QT_c_ but impoverished repolarization reserve prior to drug administration could also be at significant risk. Moreover, as female sex is itself a risk factor for drug-induced long QT syndrome [53] long-term administration of sarizotan (post-puberty) in RTT patients would need to be undertaken with care. At present, there is no alternative selective treatment for the respiratory disturbances in RTT, thus it would be premature to conclude that sarizotan is unsuitable due to its hERG-activity, particularly given the risk posed by apnoeas themselves. It is noteworthy that severe breathing dysrhythmia is independently associated with QT_c_ prolongation in RTT (OR = 2, *P* = .001) [54]. Studies in neurotypical individuals suffering from sleep apnoeas suggest a causal link [[55], [56], [57]] between frequent apnoeas and QT_c_ prolongation, but this is yet to be determined in RTT. Recent analysis suggests that extent of QT_c_ interval prolongation in RTT may vary between MECP2 mutations, with R255* mutations and large deletions being most strongly linked to QT_c_ prolongation [58]. If this is borne out by further investigations with larger sample sizes, mutation type as well as severity of respiratory arrhythmia could aid patient selection for sarizotan treatment. Additionally, evaluation of the value of other compounds with 5-HT_1A_ agonist activity as potential alternative RTT treatments [59] could usefully consider their hERG-blocking propensity (or lack thereof) alongside efficacy against respiratory dysrhythmia.

## 5 Perspectives

The results of this study demonstrate that sarizotan inhibits I_hERG_/I_Kr_ and delays ventricular repolarization at concentrations relevant to those being considered for the treatment of RTT. Given the paucity of alternative RTT treatments, if the drug is found to be beneficial for respiratory arrhythmias in RTT patients, the findings of this study indicate that treatment should commence under clinical supervision with close monitoring of ECG and electrolyte levels, with further caution to: (i) use the lowest effective dose; (ii) avoid use in patients with existing LQTS or in combination with other QT_c_ prolonging medications; and (iii) consider other risk factors for acquired LQTS [53].

## Disclosures

APA has acted as a consultant and received past research funding for unrelated projects from Neurolixis Inc.

## Appendix A Supplementary data

Supplementary material Image 1
