# Supplementary material for: Potent hERG channel inhibition by sarizotan, an investigative treatment for Rett Syndrome
Source: J Mol Cell Cardiol. 2019 Oct;135:22–30. doi: 10.1016/j.yjmcc.2019.07.012 (PMC6856717; doi:10.1016/j.yjmcc.2019.07.012)
Supplement: Supplementary file 1 — Supplementary material [file mmc1.docx]

**Online supplementary information for**

**Potent hERG channel inhibition by sarizotan, an investigative treatment for Rett Syndrome**

***by***

**Hongwei Cheng^1*^, Chunyun Du^1*^, Yihong Zhang^1^, Andrew F James^1^, Christopher E. Dempsey^2^, Ana P. Abdala^1^, Jules C. Hancox^1+,^**

^1^ School of Physiology, Pharmacology and Neuroscience, and ^2^ School of Biochemistry,

Biomedical Sciences Building, University Walk, Bristol, BS8 1TD. United Kingdom.

* These authors contributed equally to this work

^+^ jules.hancox@bristol.ac.uk

**Key words:**hERG; KCNH2; long QT syndrome*;* Rett Syndrome; sarizotan

**Figure S1: Development of I_hERG_ block during sustained depolarization**.

Ai and Aii show respectively current traces and voltage protocol used. Membrane potential was held at -80 mV and stepped to 0 mV for 10 seconds. The protocol was applied first in control solution, then discontinued whilst the cell was exposed to 100 nMsarizotan. After 3 minutes of drug exposure the protocol was applied again. Thus the “100 nMSarizotan” trace in Ai was the first record obtained following drug exposure. Fractional inhibition of I_hERG_ was calculated at regular (100 ms) intervals throughout the sustained depolarization and plotted as shown in B (mean ± SEM values from 6 similar experiments are shown).

Significant block was evident at early time-points during the protocol. The mean data in B show a trend towards a progressive increase in inhibition throughout the 10 s depolarization. However, as shown by the SEM values, there was considerable variability between results of the 6 experiments. Repeated measures ANOVA was used to compare fractional block at 100 ms, 300ms, 500 ms, 1s, and then at further 1 s intervals to 10 s into the sustained depolarization. This yielded a P value of 0.18 and thus no statistical significance in time-dependence of block was found. For completeness, pairwise comparison between the different sampled time-points was conducted using Tukey post-test and no significant differences were observed in any of the comparisons.


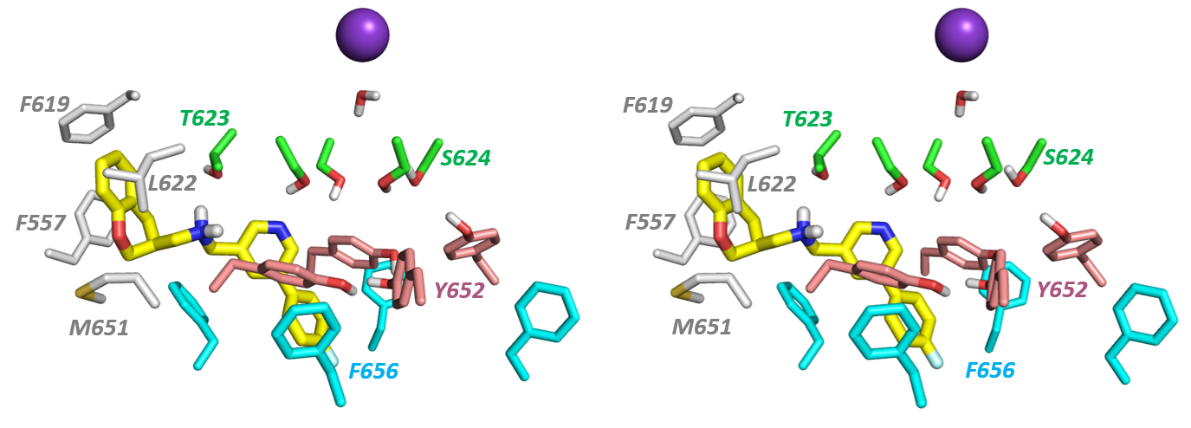


**Figure S2**: Stereo view of low energy score configuration for R-sarizotan docked into the EM structure of Wang and MacKinnon (2017).

This is the same structure and view as shown in Figure 5Ci and Cii of the main paper.

IC_50(app)_ μM fold effect δΔG_app_(kJ.mol^-1^)

F656V 1.0 5.5 -4.4

F557L 7.1 39 -9.4

S624A 5.7 31 -8.9

Y652A 5.7 31 -8.9

N588K 6.5 36 -9.2

**Table S1: Estimated IC_50_ values (IC_50(app)_) and binding free energies (δΔG_app_) for sarizotan block of hERG mutants.** The IC_50_ values are estimates (apparent IC_50_) calculated from the % block of sarizotanfrom the data in Figure 5B of the main paper (assuming a Hill coefficient of 1.0).Estimated IC_50_ is least affected by errors in the Hill coefficient at % block values near 50% and so these values were derived using appropriate % block from either 1 μM or 10 μM sarizotan, as each mutant had a % block value near 50% at one of these two concentrations (from Figure 5B). δΔG_app_ values are the apparent free energy contributions to binding from the side chain interactions lost in each of the mutants. The mutants are color-coded according to the colors in the structure figures of the main paper and supplement.
